# Feasibility and acceptability of the smart pillbox and medication label with differentiated care to support person-centered tuberculosis care among ASCENT trial participants – A multicountry study

**DOI:** 10.3389/fpubh.2024.1327971

**Published:** 2024-02-20

**Authors:** Amare W. Tadesse, Andrew Mganga, Tanyaradzwa N. Dube, Jason Alacapa, Kristian van Kalmthout, Taye Letta, Liberate Mleoh, Anna M. C. Garfin, Noriah Maraba, Salome Charalambous, Nicola Foster, Degu Jerene, Katherine L. Fielding

**Affiliations:** ^1^TB Centre, Department of Infectious Disease Epidemiology and International Health, London School of Hygiene and Tropical Medicine (LSHTM), London, United Kingdom; ^2^KNCV Tuberculosis Plus, Dar es Salaam, Tanzania; ^3^Implementation Research Division, The Aurum Institute, Johannesburg, South Africa; ^4^KNCV Tuberculosis Plus, Manila, Philippines; ^5^Evidence and Impact, KNCV Tuberculosis Plus, The Hague, Netherlands; ^6^National Tuberculosis Control Program, Ethiopian Ministry of Health, Addis Ababa, Ethiopia; ^7^National Tuberculosis Control Program, Ministry of Health, Dodoma, Tanzania; ^8^National Tuberculosis Control Program, Department of Health, Manila, Philippines; ^9^School of Public Health, University of Witwatersrand, Johannesburg, South Africa

**Keywords:** digital adherence technologies, smart pillbox, medication label, tuberculosis, acceptability, feasibility

## Abstract

**Introduction:**

Digital adherence technologies (DATs) can offer alternative approaches to support tuberculosis treatment medication adherence. Evidence on their feasibility and acceptability in high TB burden settings is limited. We conducted a cross-sectional survey among adults with drug-sensitive tuberculosis (DS-TB), participating in pragmatic cluster-randomized trials for the Adherence Support Coalition to End TB project in Ethiopia (PACTR202008776694999), the Philippines, South Africa and Tanzania (ISRCTN 17706019).

**Methods:**

From each country we selected 10 health facilities implementing the DAT intervention (smart pillbox or medication labels, with differentiated care support), ensuring inclusion of urban/rural and public/private facilities. Adults on DS-TB regimen using a DAT were randomly selected from each facility. Feasibility of the DATs was assessed using a standardized tool. Acceptability was measured using a 5-point Likert-scale, using the Capability, Opportunity, Motivation, Behavior (COM-B) model. Mean scores of Likert-scale responses within each COM-B category were estimated, adjusted for facility-level clustering. Data were summarized by country and DAT type.

**Results:**

Participants using either the pillbox (*n* = 210) or labels (*n* = 169) were surveyed. Among pillbox users, phone ownership (79%), use of pillbox reminders (87%) and taking treatment without the pillbox (22%) varied by country. Among label users, phone ownership (81%), paying extra to use the labels (8%) and taking treatment without using labels (41%) varied by country. Poor network, problems with phone charging and access, not having the pillbox and forgetting to send text were reasons for not using DATs. Overall, people with TB had a favorable impression of both DATs, with mean composite scores between 4·21 to 4·42 across COM-B categories. Some disclosure concerns were reported.

**Conclusion:**

From client-perspective, pillboxes and medication labels with differentiated care support were feasible to implement and acceptable in variety of settings. However, implementation challenges related to network, phone access, stigma, additional costs to people with TB to use DATs need to be addressed.

## Introduction

Poor adherence to tuberculosis (TB) medication has been linked to increased risk of poor treatment outcomes, including treatment failure, relapse, and development of drug resistance (1). Several strategies have been designed and implemented to monitor and improve adherence to tuberculosis medications, including community- or home-based or facility-based Directly Observed Treatment (DOT) and digital medication monitors (2). While DOT entails supervision of the administration of treatment by a trained observer watching the person with TB swallow the anti-TB medications, self-administered treatment does not involve any form of supervision on administration of treatment. Although DOT is a widely used means of supporting people on TB treatment (2), it has been criticized as it assumes that all people with TB require the same level of monitoring and support. DOT requires constant supervision of medication intake, which can pose logistical challenges and be resource-intensive for healthcare systems, especially in areas with limited resources or geographical constraints. This approach may not consider individual variations in the needs of people with TB or their responsiveness to treatment, placing a significant burden on both people with TB and health systems (3–6). Digital adherence technologies (DATs), such as smart pillboxes and text messaging, offer alternative approaches to support person-centered, differentiated care and improve treatment adherence. However, evidence on the feasibility and acceptability of such technologies is limited in high TB burden settings.

Healthcare interventions require actions from multiple sets of actors to implement successfully, and therefore feasibility and acceptability of the intervention have become key factors in the design, implementation and evaluation of interventions (7, 8), including DATs. Previous research has underscored that if an intervention is considered acceptable from clients' perspectives, clients are more likely to adhere to treatment recommendations and to benefit from improved treatment outcomes (9, 10). Furthermore, it is important to understand the feasibility of the interventions as it may influence client acceptability and/ or the desired mode of delivery of the interventions which ultimately may have an impact on the overall effectiveness of the intervention.

Despite global recommendations to use digital adherence technologies (DATs) to support tuberculosis (TB) treatment adherence, implementation continues to vary across countries and settings (11–13). Contextual factors, including socio-demographic, person-related factors, and physical and social environments, may also operate differently on the feasibility or acceptability of DATs to deliver the targeted treatment support that is needed. DATs and associated differentiated care are being assessed in many evaluations including the Adherence Support Coalition to End TB (ASCENT) cluster randomized trials (CRTs), which are being conducted in five countries with varying epidemiology, socioeconomic, geographical, infrastructural and health system factors (14, 15). We report findings on feasibility and acceptability of DATs with differentiated response to treatment adherence among adult participants enrolled into CRTs in four countries implementing the ASCENT project.

## Methods

### Study design and setting

This was a cross-sectional survey among adults with drug-sensitive tuberculosis (DS-TB) who participated in the ASCENT CRTs in Ethiopia, the Philippines, South Africa, and Tanzania. The design of the trials has been described elsewhere (14, 15). The Ethiopian trial was registered at Pan African Clinical Trials Registry (PACTR202008776694999) and the trials in the other countries were registered at International Standard Randomized Controlled Trial Number (ISRCTN 17706019).

The trials were conducted in health facilities randomized to either of the two DAT (the smart pillbox or the medication label) intervention arms or the standard of care arm (14, 15). In pillbox implementing facilities, the TB care provider offered participants a smart pillbox (evrimed 1000c, manufactured by Wisepill Technologies, South Africa) that had a configurable audio-visual reminder at a pre-defined time based on participants' preference. TB medication and dosing instructions were placed inside the pillbox. When participants opened the pillbox, a signal was sent in real-time to automatically log their daily dose to a web-based platform (Everwell Hub) via internet connection. Participants were given the pillbox within the first 4 weeks of starting treatment, expected to use it for their 6-month treatment course and informed to return the pillbox at the time of TB treatment completion. In medication labels implementing facilities, participants were given their blister-packaged TB medication with a study-customized label containing a code placed on top of the package. At the time of taking their treatment, participants were expected to text this code daily, which was automatically logged onto the web-based platform. The DAT engagement data on the platform were reviewed by TB care providers (interns provided support to TB care providers in South Africa) to evaluate daily dosing and offer differentiated care, and included automated reminder text messages, phone calls or home visit, as appropriate (14, 15). A pre-requisite for the labels intervention was the participant having access to a mobile phone. In the absence of phone access, they were offered the pillbox instead.

### Participants and eligibility

For the survey, in each country, 10 health facilities implementing the DAT intervention (five implementing the pillbox and five implementing labels intervention) were selected aiming to ensure inclusion of urban/rural and public/private facilities, and data collected from 6 to 12 participants per facility who had started a DAT. Participant selection was random, aiming to ensure a 1:1 male to female ratio and intensive to continuation treatment phase ratio, and was conducted between October 2021 and May 2022.

### Data collection

Participants were interviewed at the facility they were receiving care, by a researcher using a questionnaire adapted from the standardized tools previously used in a multi-country TB implementation research project to assess feasibility and acceptability of DATs (16, 17). Measures were grouped into indicators of feasibility and acceptability of the DAT, from the participant perspective. Feasibility was measured using indicators of actual use/experience of the DAT intervention, including access to mobile phones, use of the pillbox reminder, ever taking TB medicine without using the DAT and paying extra to use the DAT. In addition, being contacted by health care worker for a missed dose and being shown participants' adherence profile were used to assess feasibility of the differentiated care. Acceptability of using the DAT was assessed using a multi-item questionnaire (14 items for pillbox users and 12 items for labels users) with responses to each question based on a five-point Likert scale ranging from “strongly disagree” to “strongly agree” (corresponding to a scale 1 to 5).

### Data analysis

Measures to assess acceptability focused on selected domains of the Theoretical Domains Framework (TDF) of behavior change that are related to Capability (knowledge, attention, memory, and decision processes necessary to use DAT), Opportunity (social and environmental context conducive to using DAT) and Motivation (optimism, reinforcement, and emotion to want to use DAT) of performing a behavior (COM-B) (18–20). Accordingly, capability, opportunity and motivation to use either pillbox or labels were assessed separately. We also reported capability, opportunity and motivation by sex, age group (<40, ≥40 years), treatment phase (intensive, continuation) and area of residence (urban, rural). In addition, we did a *post-hoc* analysis comparing COM-B domains for participants attending private versus public facilities.

Feasibility and acceptability indicators were summarized by DAT type and country. For Likert scales, we summarized mean scores and 95% confidence intervals, adjusting for clustering at the facility-level using robust standard errors. If agreement to items indicated unfavorable impression of DAT, responses were reverse coded to reflect favorable impression of DAT prior to analysis. Composite scores for capability, opportunity and motivation were calculated for each participant by using the mean response to Likert scale questions within each category. For individual components, we reported the percentage of agreement, by grouping strongly agree and agree categories. Participants were analyzed based on the DAT they received at the time of interview. Data were analyzed using Stata version 16.0.

## Results

Of 379 participants interviewed across the four countries, 210 (55.4%) participants were using the pillbox in 24 health facilities and 169 (44.6) were using medication labels in 20 health facilities (Table 1). Four facilities randomized to the labels intervention (one in the Philippines and three in Tanzania) included participants who were using the pillbox because of lack of cell phone access. One facility in South Africa randomized to the labels arm had switched enrolment to the pillbox arm at the time of the sub-study and only enrolled participants using the pillbox.

**Table 1:** Characteristics of study participants, overall and country, by type of DAT (*n* = 379).

	**All countries (*n =* 379)**		**Ethiopia (*n =* 100)**		**The Philippines (*n =* 102)**		**South Africa (*n =* 78)**		**Tanzania (*n =* 99)**	
	**Pillbox**	**Labels**	**Pillbox**	**Labels**	**Pillbox**	**Labels**	**Pillbox**	**Labels**	**Pillbox**	**Labels**
**Individual level**										
DAT received	210	169	50	50	50	52	49	29	61	38
Age (years), median, IQR	41 (30, 53)	36 (27, 50)	35 (24, 44)	33.5 (24,39)	45 (32, 56)	38 (26, 53)	43 (34, 52)	41 (31, 47)	42 (35, 52)	40.5 (34, 56)
Female, *n* (%)	95 (45%)	73 (43%)	25 (50%)	25 (50%)	25 (50%)	26 (52%)	19 (39%)	8 (28%)	26 (42%)	14 (37%)
HIV status positive, *n* (%)	57/157 (36%)^*^	33/117 (28%)^*^	7 (14%)	5 (10%)	NA	NA	32 (65%)	19 (66%)	18 (31%)^**^	9 (24%)
On ART (among those HIV-positive), *n* (%)	54 (95%)	32 (97%)	7 (100%)	5 (100%)	NA	NA	29 (91%)	18 (95%)	18 (100%)	9 (100%)
Continuation phase, *n* (%)	131 (62%)	119 (70%)	31 (62%)	31 (62%)	34 (68%)	44 (85%)	28 (57%)	20 (69%)	38 (62%)	24 (63%)
PTB, *n* (%)	189 (91%)^***^	159 (94%)	50 (100%)	50 (100%)	50 (100%)	50 (100%)	41 (84%)	22 (76%)	48 (81%)^***^	35 (92%)
Bacteriologic confirmed, *n* (%)	114 (55%)^***^	92 (54%)	29 (58%)	28 (56%)	26 (52%)	30 (58%)	26 (53%)	16 (55%)	33 (56%)^***^	18 (47%)
**Cluster-level**										
# facilities randomized to the DAT arm	20	20	5	5	5	5	5	5	5	5
Urban, *n* (%)	15 (75%)	13 (65%)	4 (80%)	4 (80%)	4 (80%)	3 (60%)	4 (80%)	4 (80%)	3 (60%)	2 (40%)
Facility type – Clinic: public *n* (%)	18 (90%)	19 (95%)	5 (100%)	5 (100%)	5 (100%)	4 (80%)	5 (100%)	5 (100%)	3 (60%)	5 (100%)
Data collection period	8/10/2021–25/5/2022	21/10/2021–12/5/2022	8/10/2021–20/4/2022	21/10/2021–12/5/2022	21/2/2022–25/5/2022	17/2/2022–20/4/2022	24/1/2022–25/5/2022	9/12/2021–9/3/2022	3/2/2022–14/3/2022	3/2/2022–2/3/2022

^*^Excluding the Philippines where HIV status data are not available; ^**^data missing for n = 3 participants using the pillbox; ^***^data missing for n = 2 participants using the pillbox. IQR, interquartile range; PTB, pulmonary tuberculosis; NA, not available.

Among participants using the pillbox, the median age was 41 years (interquartile range [IQR]: 30, 53) with relatively younger participants from Ethiopia; 45% were female (lower in South Africa, 39%); 36% were living with HIV (highest in South Africa, 65%); and 66% were in the continuation phase of their TB treatment course. Participants using the labels were younger with median (IQR) age of 36 years (27, 50), 43% were female (lower in South Africa, 28%), 28% HIV co-infected with South Africa having the highest co-infection, 66%; and 70% were in the continuation phase of their TB treatment course (higher in the Philippines, 85%) (Table 1).

### Feasibility of smart pillbox intervention

Mobile phone access was not required for participants using the pillbox though is necessary to implement components of the differentiated care. Most participants using the pillbox had their own mobile phone (79%; 165/210), either not shared or were the primary owner of a shared phone, with some variation by country. Phone ownership was slightly higher among participants aged <40 years (85%, 82/97) compared with their counterparts (73%, 83/113). Owning a mobile phone which was not shared was most common in South Africa (90%; 44/49) and least common in the Philippines (62%; 31/50). Changing phone number/SIM card in the last year was most common in the Philippines (26%; 13/50) and Tanzania (23%; 14/61).

The majority (87%; 183/210) of participants reported use of pillbox reminder to take TB medicine, though participants in Ethiopia reported relatively lower use (73%; 36/50) than the other countries, with 34% (17/50) reporting their family/ friends reminding them to take their medicine. At least 90% of participants, similar by country, reported that their households' members knew about their pillbox use. Overall, 71% of participants reported being shown their adherence information by a health care worker, the lowest percentage being in the Philippines (46%; 23/50) and highest in South Africa (94%; 46/49). Among 25 participants who reported a missed dose (12%; 25/210), 9 (36%) reported no subsequent contact by health care worker (with the highest in the Philippines, 78%).

Taking TB medicine without the pillbox varied by country, being most common in the Philippines (38%; 19/50) and least common in Tanzania (10%; 6/61). The most common reason was not having the pillbox with them. Poor network connection and charging problems were also reasons reported by participants from the Philippines and not wanting to be seen using the pillbox, reported only in South Africa (Table 2).

**Table 2:** Feasibility of DAT interventions, by country and DAT type.

**Pillbox**	**All (*n =* 210)**	**Ethiopia (*n =* 50)**	**The Philippines (*n =* 50)**	**South Africa (*n =* 49)**	**Tanzania (*n =* 61)**
Mobile phone access, *n* (%)	200 (95.2%)	48 (96%)	48 (96.0%)	47 (95.9%)	57 (93.4%)
Own mobile phone and not shared, *n* (%)	154 (73.3%)	36 (72%)	31 (62%)	44 (89.8%)	43 (70.5%)
Own mobile and share with family, *n* (%)	11 (5.2%)	0 (0%)	5 (10.0%)	0 (0%)	6 (9.8%)
Family shared phone, not own, *n* (%)	35 (16.7%)	12 (24%)	12 (24%)	3 (6.1%)	8 (13.1%)
Changed phone number/ SIM in the last 1 year, *n* (%)	33 (15.7%)	3 (6.0%)	13 (26%)	3 (6.1%)	14 (23%)
Use box alert to remind to take TB medicine, *n* (%)	183 (87.1%)	36 (72%)	43 (86%)	49 (100%)	55 (90.2%)
Household members know participant uses box, *n* (%)	198 (94.3%)	48 (96%)	44 (88%)	46 (93.9%)	60 (98.4%)
Ever missed a dose, *n* (%)	25 (11.9%)	3 (6.0%)	9 (18%)	9 (18.4%)	4 (6.6%)
No contact by health care worker for a missed dose % (n/N)^a^	9 (36%)	0 (0%)	7 (77.8%)	1 (11.1%)	1 (25%)
Paid extra to use DAT, *n* (%)	11 (4.8%)	0 (0%)	4 (6.8%)	0 (0%)	7 (11.3%)
phone credit ^b^	10 (90.9)	0 (0%)	3 (75.0%)	0 (0%)	7 (100%)
Health care worker showed adherence information, *n* (%)	150 (71.4%)	32 (64.0%)	23 (46%)	46 (93.9%)	49 (80.3%)
Ever taken TB medicine without using box, *n* (%)	45 (22%)	11 (22.0%)	19 (38%)	9 (18.4%)	6 (9.8%)
Not having the box with them^c^	31 (68.9%)	8 (72.7%)	10 (52.6%)	8 (88.9%)	5 (83.3%)
Poor network connection^c^	8 (17.8%)	1 (9.0%)	7 (36.8%)	0 (0%)	0 (0%)
Did not want to be seen using box^c^	1 (2.2%)	0 (0%)	0 (0%)	1 (11.1%)	0 (0%)
Charging problems^c^	3 (6.7%)	0 (0%)	3 (15.8%)	0 (0%)	0 (0%)
**Medication labels**	**All (***n =* **169)**	**Ethiopia (***n =* **50)**	**The Philippines (***n =* **52)**	**South Africa (***n =* **29)**	**Tanzania (***n =* **38)**
Phone access, *n* (%)	168 (99.4%)	50 (100%)	52 (100%)	29 (100%)	37 (97.4%)
Own mobile phone, *n* (%)	122 (72.2%)	35 (70.0%)	33 (63.5%)	28 (96.6%)	26 (68.4%)
Own mobile and share with family, *n* (%)	14 (8.3%)	2 (4.0%)	10 (19.2%)	1 (3.4%)	1 (2.6%)
Family shared phone, not own, *n* (%)	32 (18.9%)	13 (26.0%)	9 (17.3%)	0 (0%)	10 (26.3%)
Changed phone number/ SIM in the last 1 year, *n* (%)	35 (20.7%)	7 (14.0%)	11 (21.2%)	11 (37.9%)	6 (15.8%)
Set alarm to remind to take TB medicine, *n* (%)	41 (24.3%)	7 (14%)	17 (32.7%)	14 (48.3%)	3 (7.9%)
Family/ friend remind to take TB medicine, *n* (%)	69 (40.8%)	34 (68.0%)	20 (38.5%)	4 (13.8%)	11 (28.9%)
Household members know participant uses label, *n* (%)	160 (94.7%)	49 (98.0%)	50 (100%)	24 (82.9%)	37 (97.4%)
Ever missed a dose, *n* (%)	22 (13.0%)	2 (4.0%)	9 (17.3%)	7 (24.1%)	4 (10.5%)
No contact by health care worker for a missed dose %, (n/N)^a^	7 (31.8%)	0 (0%)	6 (66.7%)	1 (14.3%)	0 (0%)
Paid extra to use DAT, *n* (%)	15 (8.1%)	3 (6.0%)	3 (5.8%)	2 (6.9%)	7 (18.4%)
Phone credit^b^	8 (53.3%)	0 (0%)	3 (100%)	1 (50.0%)	4 (57.1%)
New phone^b^	5 (33.3%)	1 (33.3%)	0 (0%)	2 (100%)	2 (28.6%)
Repair phone^b^	2 (28.6%)	2 (66.7%)	0 (0%)	0 (0%)	0 (0%)
Health care worker showed adherence information, *n* (%)	114 (67.5%)	35 (70.0%)	23 (44.2%)	27 (93.1%)	29 (76.3%)
Ever taken TB medicine without using label, *n* (%)	70 (41.4%)	16 (32.0%)	27 (51.9%)	15 (51.7%)	12 (31.6%)
Phone access challenges^c^	19 (27.1%)	7 (43.8%)	4 (14.8%)	3 (20.0%)	5 (41.7%)
Poor network connection^c^	10 (14.3%)	1 (6.3%)	8 (29.6%)	0 (0%)	1 (8.3%)
Forgot to send text^c^	40 (57.1%)	7 (43.8%)	20 (74%)	8 (53.3%)	5 (41.7%)
Charging problems^c^	8 (11.4%)	3 (18.8%)	2 (7.4%)	2 (13.3%)	1 (8.3%)

^a^% out of those who reported a missed dose (non-engagement with the technology); ^b^% out of those who paid extra to use the DAT; ^c^% out of those who reported taking treatment without using pillbox.

### Feasibility of medication label intervention

The majority of participants owned their phone (80%; 136/169), though this varied by country (71% in Tanzania to 100% in South Africa). Phone ownership appeared to be more common among participants aged <40 years (83%, 80/96) compared with their counterparts (77%, 56/73). Reminders to take their TB medication included family/friends (41%; 69/169), most common in Ethiopia (68%) and least common in South Africa (14%), and use of an alarm (24%; 41/169), common in the Philippines (33%) and South Africa (48%).

Overall, 13% (22/169) reported ever missing a dose which varied by country: lowest in Ethiopia (4%; 2/50) and highest in South Africa (24%; 7/29). Of those who reported missing a dose, one-third (32%; 7/22) reported no subsequent contact with a health care worker (with the highest in the Philippines, 67%). Two-thirds (68%, 114/169) reported being shown their adherence information by a health care worker; lowest in the Philippines (44%; 23/50) and highest in South Africa (93%; 27/29). Fifteen participants (8%) reported to have paid extra to use the medication label, mainly for phone credit and buying a new phone. Overall, 41% (70/169) reported to have ever taken their TB medicine without using the label (not texting back to the toll-free number) which was most common among participants from the Philippines (52%; 27/52) and South Africa (52%; 15/29). The reasons included forgetting to send text (57%), challenges in daily phone access (27%), poor connectivity (14%) and charging problems (11%), which varied by country (Table 2).

### Acceptability of smart pillbox and labels interventions

**Figure 1:**
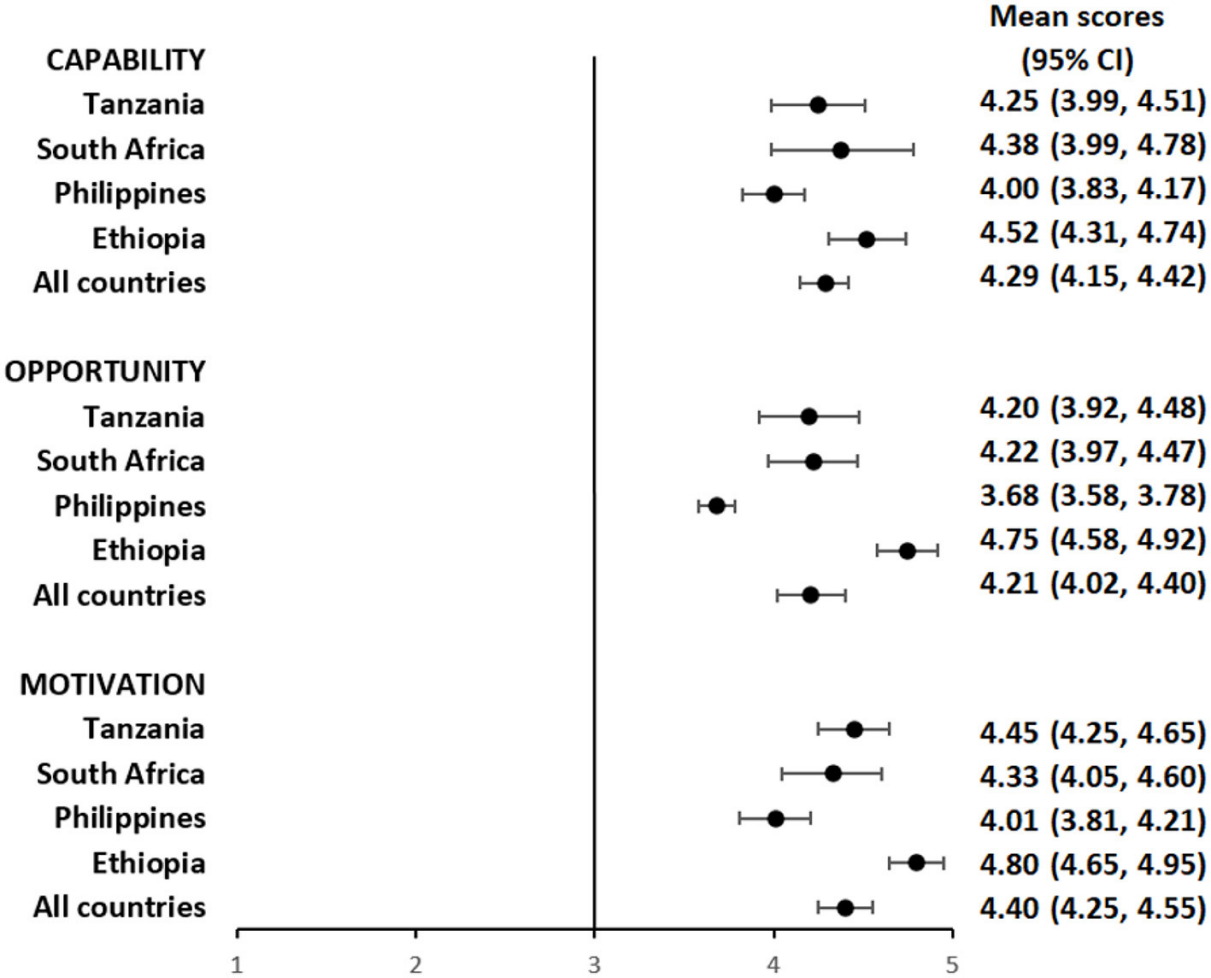
Mean capability, opportunity, and motivation scores by country among people with TB using pillbox.

**Figure 2:**
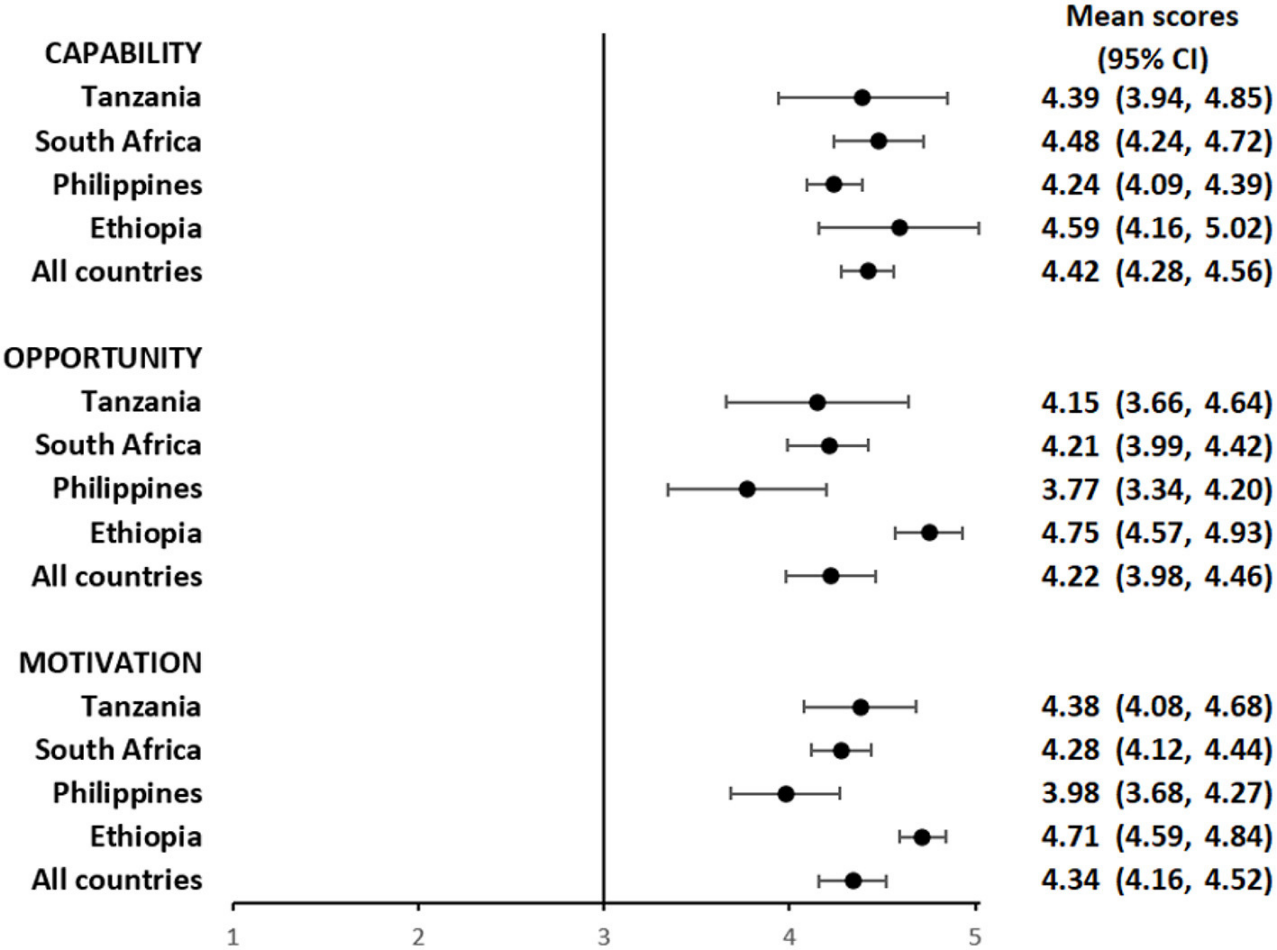
Mean capability, opportunity, and motivation scores by country among people with TB using medication labels.

Overall, participants had a favorable impression of their capability to use the pillbox or the labels. The mean composite scores for questions focusing on capability among pillbox users was 4.29 (95%CI: 4.15, 4.42) (Figure 1 and Supplementary Table S1) and label users was 4.42 (95%CI: 4.28, 4.56) (Figure 2 and Supplementary Table S2), reflecting a response between agree and strongly agree.

The mean composite scores for questions related to opportunity among pillbox users was 4.21 (95%CI: 4.02, 4.40) (Figure 1 and Supplementary Table S1) and label users was 4.22 (95%CI: 3.98, 4.46) (Figure 2 and Supplementary Table S2) suggesting that both DATs were acceptable. However, there were some stigma-related concerns related to using the DATs, especially being uncomfortable using the DAT in front of other people (agree/strongly agree: pillbox 20%, 42/210 and labels 18%, 30/169), worried that using DAT may lead to disclosure of TB status (agree/strongly agree: pillbox 21%, 44/210 and labels 18%, 31/169) and being uncomfortable using the DAT outside home, including at work or travel (agree/strongly agree: pillbox 20%, 41/210 and labels 17%, 29/169).

For motivation, the mean composite score among pillbox users was 4.40 (95%CI: 4.25, 4.55) (Figure 1 and Supplementary Table S1) and label users was 4.34 (95%CI: 4.16, 4.52) (Figure 2 and Supplementary Table S2). Nearly all participants reported that using DATs motivated them to complete their TB treatment and get healthy while there were some concerns about data privacy from participants in the Philippines and South Africa.

The average capability, opportunity and motivation scores for both DAT types was lowest in the Philippines and highest in Ethiopia (Figures 1, 2). There were no differences in mean capability, opportunity and motivation scores by sex, age, treatment phase and area of residence for each DAT type (Supplementary Tables S3a, b, S4a, b). In Tanzania and the Philippines, there were 20 participants (all pillbox) and 10 participants (all labels) attending private facilities, respectively. Mean scores for COM-B domains were similar by facility type (Supplementary Table S5).

## Discussion

In this study conducted as part of the ASCENT trials in Ethiopia, the Philippines, South Africa and Tanzania, we found that the smart pillbox and the medication labels were generally both feasible to implement and acceptable to people with TB, with some differences by country and DAT-type observed. These differences included, a greater proportion of people with TB owning their own mobile phones in South Africa compared to the Philippines. In Ethiopia, people with TB reported that they used reminders from friends or family in addition to the use of the pillbox. Structural challenges related to poor mobile network connectivity and power outages contributed to the decreased operational feasibility of DATs. People with TB using DATs also expressed concerns around stigma and privacy issues when using DATs. We found no differences in mean capability, opportunity and motivation scores between groups of gender, age, treatment phase and area of residence on acceptability of the two DAT types.

Our findings highlight the importance of contextual adaptations, with a focus on TB care provider's role in client differentiation and monitoring adherence to provide timely, tailored adherence support, prior to implementations of DAT. These may include using medication labels for those with good access to phones who reported any inconvenience in carrying the pillbox, availing affordable phones for the duration of treatment to ensure daily access to phones and engaging other health staff in the TB clinics to support the delivery of the differentiated care. The literature has provided evidence indicating that the act of participants opening a smart pillbox may not necessarily correlate with the actual ingestion of their TB medication (12), though this has not been reported in our study. Interventions aimed at improving medication adherence should follow a holistic approach that goes beyond monitoring pillbox openings. Addressing individual behavioral and psychosocial factors may be necessary for enhanced effectiveness.

Technological fatigue could partly explain issues of forgetting to send confirmation messages by participants using medication labels or not having the pillbox with them when taking TB medicine. Strategies such as family or caregiver involvement in reminding and supporting people with TB and provision of thorough training for people with TB on how to use the DATs and offering ongoing support could address these challenges. Technology fatigue could result in the participant discontinuing the use of the DAT, although this was not observed in this cross-sectional survey.

Good access to mobile phones, enabling early notification and response through text message or phone calls to missed doses provided encouraging indications of the feasibility of both DAT interventions. However, shared phones and changing phone numbers/SIM cards, paying extra credit, poor network, charging problems posed challenges to the feasibility of implementing the DAT interventions. In Tanzania and the Philippines, a toll-free number still required phone credit. Results from a meta-analysis and systematic review of DATs and other studies have also documented barriers to the feasibility of implementing smart pillboxes and SMS-style interventions, such as technical issues with phones, network connectivity and issues with charging phones/pillboxes in different contexts (12, 17, 21, 22).

Acceptability was evidenced by high capability, opportunity, and motivation to use the smart pillbox and medication labels among people with TB. Our findings support previous studies in Sub-Saharan Africa and Asia which demonstrated electronic medication monitors and SMS-style interventions offer a feasible and acceptable person-centered differentiated TB care (11, 13, 17, 21, 23–27). However, fear of stigma related to taking TB medication using the DATs and disclosure of one's TB status contributed to lower opportunity scores, highlighting the influence of stigma on TB care (28). These may limit the use of the pillbox and could also partly explain why most participants who have ever taken their TB medicine without the pillbox reported that they did not have the pillbox with them while traveling or going to work. Furthermore, when advocating for the use of Digital Adherence Technologies (DATs), especially those involving SMS reminders, policymakers and implementers should be mindful of privacy considerations. Obtaining consent for the use of DATs and receiving reminders, avoiding the inclusion of sensitive information in SMS reminders, and restricting access to client data to authorized healthcare professionals are potential approaches to protect the confidentiality and wellbeing of individuals undergoing TB treatment.

The composite mean scores for capability, opportunity and motivation were highest in Ethiopia and lowest in the Philippines. A recent meta-analysis of implementation of DATs in Sub-Saharan Africa and Asia reported relatively higher scores for the same domains in the Philippines (17). The observed differences between the studies could be due to the differences in study sites with the meta-analysis including experiences from implementation in private health facilities only, and survey was not conducted by an independent research team.

Key strengths of our study included that it was part of a large pragmatic cluster randomized trial conducted in four countries with different geographical, technological and socioeconomical contexts that would support generalizability of findings to trial participants across the countries. We evaluated acceptability of DATs using a Theoretical Domains Framework that enabled assessment of implementation of DATs. Thirdly, selection of DAT was randomized by clusters which, to some extent, allowed direct comparison of the two DAT types. However, interpretation of findings should be done cautiously as the study has some limitations. The study was conducted on a sub-sample of the trial participants, with a small sample size per country, limited data from private facilities which may not represent all people with TB in the study countries. Social desirability bias or inclusion of more participants with good adherence may result in more favorable responses. Perspectives of health care workers and other stakeholders on DAT was not assessed.

In conclusion, we found that using smart pillboxes or medication labels DATs with differentiated care to support TB care was feasible to implement and acceptable from client perspective. However, implementation and DAT-specific challenges, including power outages, poor network, fear of stigma, not using pillbox during travel and situation that necessitates client incurred additional costs need to be addressed. Qualitative studies to enhance better understanding of the facilitators and barriers to DAT feasibility and acceptability are recommended. Effectiveness of these DATs to improve treatment outcomes, including cost effectiveness of the interventions should be further explored.

## Data availability statement

The raw data supporting the conclusions of this article will be made available by the authors, without undue reservation.

## Ethics statement

The studies involving humans were approved by the London School of Hygiene and Tropical Medicine Ethics Committee (19120 and 19135), United Kingdom; WHO Ethical Review Committee (ERC.0003297 and 0003296), Switzerland; Individual country-specific ethics committees: Ethiopia [Addis Ababa City Administration Health Bureau Public Emergency and Health Research Directorate Institutional Review Board (AA16238/227) and Oromia Regional Health Bureau Public Emergency and Health Research Directorate Institutional Review Board (BEFO/HBTFH/1–16/10415)], Philippines (Single Joint Research Ethics Board SJREB 2019–57), South Africa (University of Witwatersrand Human Research Ethics Committee AUR2–1–268), and Tanzania [Tanzania Medical Research Coordinating Committee (MRCC) at National Institute for Medical Research, Dar es Salaam NIMR/HQ/R.8a/Vol.IX/3431] have approved the main trial and the sub-study. The studies were conducted in accordance with the local legislation and institutional requirements. Written informed consent for participation in this study was provided by the participants.

## Author contributions

AT: Conceptualization, Data curation, Formal analysis, Investigation, Methodology, Software, Supervision, Validation, Writing—original draft, Writing—review & editing. AM: Data curation, Project administration, Supervision, Validation, Writing—review & editing. TD: Data curation, Project administration, Supervision, Validation, Writing—review & editing. JA: Data curation, Project administration, Supervision, Validation, Writing—review & editing. KK: Conceptualization, Funding acquisition, Project administration, Resources, Supervision, Writing—review & editing. TL: Resources, Supervision, Validation, Writing—review & editing. LM: Resources, Supervision, Validation, Writing—review & editing. AG: Resources, Supervision, Validation, Writing—review & editing. NM: Data curation, Supervision, Validation, Writing—review & editing. SC: Writing—review & editing, Conceptualization, Project administration, Resources, Supervision, Validation. NF: Investigation, Methodology, Writing—review & editing. DJ: Conceptualization, Funding acquisition, Investigation, Methodology, Supervision, Writing—review & editing. KF: Conceptualization, Formal analysis, Investigation, Methodology, Project administration, Resources, Software, Supervision, Validation, Writing—review & editing.
