# Supplementary material for: Feasibility and acceptability of the smart pillbox and medication label with differentiated care to support person-centered tuberculosis care among ASCENT trial participants – A multicountry study
Source: Front Public Health. 2024 Feb 20;12:1327971. doi: 10.3389/fpubh.2024.1327971 (PMC10913790; doi:10.3389/fpubh.2024.1327971)
Supplement: Supplementary file 1 [file Data_Sheet_1.pdf]

Supplementary tables

Table S1: Mean items scores for Capability, Opportunity, and Motivation by country among people with TB using pillbox.

	Overall (n=210)		Ethiopia (n=50)		Philippines (n=50)		South Africa (n=49)		Tanzania (n=61)	
	Mean	(95% CI)	Mean	(95% CI)	Mean	(95% CI)	Mean	(95% CI)	Mean	(95% CI)
**Capability**										
I know how to use the adherence technology	4.46	(4.29, 4.63)	4.90	(4.78, 5.02)	4.24	(3.94, 4.54)	4.37	(3.79, 4.94)	4.34	(4.08, 4.61)
The reminders I receive on my phone help me to remember to take my medicine.	3.97	(3.78, 4.16)	4.06	(3.60, 4.52)	3.56	(3.23, 3.89)	4.33	(4.11, 4.54)	3.98	(3.56, 4.41)
The reminders I receive on the medication box help me to remember to take my medicine.	4.43	(4.27, 4.58)	4.62	(4.15, 5.09)	4.2	(3.75, 4.65)	4.46	(3.97, 4.94)	4.42	(4.20, 4.66)
**Opportunity**										
I am more connected to my healthcare provider while using my adherence technology	4.38	(4.18, 4.58)	4.94	(4.87, 5.00)	3.98	(3.80, 4.16)	4.20	(3.72, 4.68)	4.39	(4.05, 4.73)
I am comfortable using my adherence technology in front of other people.	3.91	(3.62, 4.20)	4.66	(4.11, 5.21)	3.44	(3.18, 3.70)	4.20	(3.94, 4.47)	3.44	(2.83, 4.05)
I am NOT worried that using the adherence technology will make it more likely that others will find out that I have TB.	3.86	(3.52, 4.20)	4.72	(4.07, 5.37)	2.96	(2.44, 3.48)	3.96	(3.57, 4.35)	3.82	(3.31, 4.33)
It DOES NOT take me too much time to take my TB medicine and open the box every day.	4.35	(4.18, 4.53)	4.88	(4.66, 5.10)	4.16	(3.87, 4.45)	4.06	(3.89, 4.23)	4.31	(3.99, 4.64)
I will recommend using adherence technologies to my family or friends if they have TB.	4.57	(4.44, 4.71)	4.88	(4.68, 5.08)	4.22	(3.91, 4.53)	4.59	(4.34, 4.84)	4.59	(4.41, 4.77)
I am comfortable using the adherence technologies outside of my home (for example at work or when I travel).	4.02	(3.71, 4.34)	4.42	(4.26, 4.58)	2.98	(2.41, 3.55)	4.29	(3.98, 4.59)	4.34	(3.77, 4.92)
I am comfortable taking the medication box to the clinic during my follow-up visit	4.38	(4.21, 4.55)	4.76	(4.62, 4.90)	4.00	(3.76, 4.24)	4.24	(3.93, 4.56)	4.47	(4.08, 4.87)
**Motivation**										
Using my adherence technology will help me complete my TB treatment.	4.55	(4.44, 4.67)	4.78	(4.54, 5.02)	4.34	(4.04, 4.64)	4.55	(4.26, 4.84)	4.54	(4.36, 4.72)
Using adherence technologies while taking TB medicines will help me get healthy.	4.49	(4.35, 4.63)	4.84	(4.65, 5.03)	4.26	(3.90, 4.62)	4.47	(4.19, 4.75)	4.41	(4.19, 4.63)
The instructions on the adherence technology makes it easy for me to remember what to do.	4.38	(4.24, 4.51)	4.66	(4.49, 4.83)	4.10	(3.91, 4.29)	4.33	(3.99, 4.65)	4.41	(4.13, 4.69)
I am NOT concerned about the privacy of my health information collected by the adherence technology	4.18	(3.90, 4.46)	4.92	(4.76, 5.08)	3.34	(3.02, 3.66)	3.96	(3.66, 4.26)	4.44	(4.11, 4.77)

Table S2: Capability, Opportunity, and Motivation scores by country among people with TB using medication labels

	Overall (n=169)		Ethiopia (n=50)		Philippines (n=52)		South Africa (n=29)		Tanzania (n=38)	
	Mean	(95% CI)	Mean	(95% CI)	Mean	(95% CI)	Mean	(95% CI)	Mean	(95% CI)
**Capability**										
I know how to use the adherence technology	4.41	(4.21, 4.62)	4.70	(4.07, 5.33)	4.19	(3.97, 4.42)	4.46	(4.22, 4.71)	4.29	(3.68, 4.90)
The reminders I receive on my phone help me to remember to take my medicine.	4.43	(4.27, 4.58)	4.48	(3.89, 5.07)	4.28	(3.99, 4.59)	4.48	(4.20, 4.77)	4.50	(4.18, 4.82)
**Opportunity**										
I am more connected to my healthcare provider while using my adherence technology	4.34	(4.11, 4.57)	4.82	(4.57, 5.07)	3.90	(3.47, 4.33)	4.27	(3.74, 4.81)	4.34	(3.91, 4.77)
I am comfortable using my adherence technology in front of other people.	4.16	(3.86, 4.45)	4.72	(4.40, 5.04)	3.79	(3.08, 4.50)	4.24	(3.86, 4.62)	3.87	(3.19, 4.54)
I am NOT worried that using the adherence technology will make it more likely that others will find out that I have TB.	3.97	(3.57, 4.37)	4.70	(4.38, 5.02)	3.25	(2.19, 4.31)	4.04	(3.51, 4.56)	3.95	(3.09, 4.81)
It DOES NOT take me too much time to take my TB medicine and text for the labels every day.	4.18	(3.92, 4.45)	4.80	(4.54, 5.06)	3.94	(3.54, 4.34)	3.83	(3.38, 4.28)	3.97	(3.29, 4.65)
I will recommend using adherence technologies to my family or friends if they have TB.	4.59	(4.38, 4.80)	4.96	(4.89, 5.03)	4.23	(3.66, 4.81)	4.55	(4.25, 4.86)	4.63	(4.38, 4.88)
I am comfortable using the adherence technologies outside of my home (for example at work or when I travel).	4.08	(3.73, 4.43)	4.52	(4.04, 4.99)	3.50	(2.33, 4.67)	4.31	(4.18, 4.44)	4.13	(3.63, 4.64)
**Motivation**										
Using my adherence technology will help me complete my TB treatment.	4.48	(4.31, 4.64)	4.68	(4.48, 4.88)	4.27	(3.73, 4.81)	4.50	(4.32, 4.68)	4.47	(4.15, 4.80)
Using adherence technologies while taking TB medicines will help me get healthy.	4.47	(4.29, 4.65)	4.74	(4.44, 5.04)	4.23	(3.73, 4.73)	4.34	(4.22, 4.47)	4.53	(4.25, 4.81)
The instructions on the adherence technology makes it easy for me to remember what to do.	4.37	(4.22, 4.51)	4.68	(4.37, 4.99)	4.12	(4.01, 4.22)	4.28	(4.01, 4.55)	4.37	(4.13, 4.61)
I am NOT concerned about the privacy of my health information collected by the adherence technology	4.05	(3.69, 4.40)	4.76	(4.35, 5.17)	3.31	(2.82, 3.79)	4.00	(3.60, 4.40)	4.16	(3.71, 4.61)

Table S3a: Mean Capability, Opportunity, and Motivation scores by sex, age and treatment phase among particiapants using the pillbox

	Sex				Age, grouped			
	Female (n=95)		Male (n=115)		<40 years (n=97)		≥40 years (n=113)	
	Mean	95% CI	Mean	95% CI	Mean	95% CI	Mean	95% CI
**Capability**	**4.23**	**(4.08, 4.39)**	**4.33**	**(4.18, 4.49)**	**4.31**	**(4.12, 4.50)**	**4.27**	**(4.14, 4.39)**
I know how to use the adherence technology	4.39	(4.21, 4.57)	4.51	(4.32, 4.71)	4.45	(4.20, 4.71)	4.46	(4.31, 4.60)
The reminders I receive on my phone help me to remember to take my medicine.	3.93	(3.69, 4.18)	4.00	(3.78, 4.22)	4.04	(3.78, 4.30)	3.91	(3.68, 4.13)
The reminders I receive on the medication box help me to remember to take my medicine.	4.36	(4.15, 4.57)	4.48	(4.30, 4.65)	4.44	(4.20, 4.68)	4.42	(4.25, 4.58)
**Opportunity**	**4.14**	**(3.93, 4.36)**	**4.27**	**(4.09, 4.45)**	**4.30**	**(4.08, 4.52)**	**4.13**	**(3.94, 4.32)**
I am more connected to my healthcare provider while using my adherence technology	4.32	(4.08, 4.55)	4.43	(4.24, 4.63)	4.52	(4.25, 4.78)	4.27	(4.08, 4.45)
I am comfortable using my adherence technology in front of other people.	3.83	(3.52, 4.14)	3.97	(3.63, 4.32)	4.05	(3.70, 4.40)	3.79	(3.46, 4.11)
I am NOT worried that using the adherence technology will make it more likely that others will find out that I have TB.	3.74	(3.31, 4.17)	3.97	(3.66, 4.27)	3.94	(3.50, 4.38)	3.80	(3.44, 4.16)
It DOES NOT take me too much time to take my TB medicine and open the box every day.	4.37	(4.19, 4.44)	4.34	(4.13, 4.55)	4.44	(4.20, 4.68)	4.28	(4.10, 4.46)
I will recommend using adherence technologies to my family or friends if they have TB.	4.53	(4.36, 4.70)	4.61	(4.47, 4.75)	4.64	(4.46, 4.81)	4.51	(4.37, 4.65)
I am comfortable using the adherence technologies outside of my home (for example at work or when I travel).	3.92	(3.53, 4.29)	4.11	(3.79, 4.43)	4.11	(3.79, 4.44)	3.95	(3.58, 4.31)
I am comfortable taking the medication box to the clinic during my follow-up visit	4.32	(4.15, 4.48)	4.43	(4.22, 4.63)	4.41	(4.21, 4.62)	4.35	(4.16, 4.53)
**Motivation**	**4.40**	**(4.26, 4.54)**	**4.40**	**(4.24, 4.56)**	**4.46**	**(4.29, 4.63)**	**4.35**	**(4.20, 4.50)**
Using my adherence technology will help me complete my TB treatment.	4.53	(4.39, 4.66)	4.57	(4.44, 4.71)	4.65	(4.53, 4.77)	4.47	(4.32, 4.62)
Using adherence technologies while taking TB medicines will help me get healthy.	4.52	(4.36, 4.67)	4.47	(4.33, 4.61)	4.58	(4.43, 4.73)	4.42	(4.27, 4.56)
The instructions on the adherence technology makes it easy for me to remember what to do.	4.32	(4.19, 4.44)	4.43	(4.25, 4.60)	4.41	(4.21, 4.61)	4.35	(4.21, 4.48)
I am NOT concerned about the privacy of my health information collected by the adherence technology	4.24	(3.96, 4.53)	4.13	(3.81, 4.44)	4.21	(3.86, 4.55)	4.16	(3.84, 4.48)

Table S3b: Mean Capability, Opportunity, and Motivation scores by sex, age and treatment phase among particiapants using the pillbox

	Treatment phase				Area of residence			
	Intensive phase (n=79)		Continuation phase (n=131)		Urban (n=151)		Rural (n=59)	
	Mean	95% CI	Mean	95% CI	Mean	95% CI	Mean	95% CI
**Capability**	**4.20**	**(4.03, 4.37)**	**4.34**	**(4.20, 4.48)**	**4.25**	**(4.07, 4.43)**	**4.37**	**(4.16, 4.58)**
I know how to use the adherence technology	4.42	(4.16, 4.68)	4.48	(4.32, 4.64)	4.40	(4.18, 4.63)	4.59	(4.34, 4.84)
The reminders I receive on my phone help me to remember to take my medicine.	3.91	(3.65, 4.17)	4.01	(3.78, 4.23)	3.97	(3.73, 4.21)	3.98	(3.57, 4.40)
The reminders I receive on the medication box help me to remember to take my medicine.	4.26	(4.03, 4.50)	4.52	(4.37, 4.68)	4.39	(4.18, 4.59)	4.53	(4.23, 4.82)
**Opportunity**	**4.15**	**(3.97, 4.34)**	**4.25**	**(4.04, 4.46)**	**4.21**	**(3.97, 4.45)**	**4.21**	**(3.84, 4.57)**
I am more connected to my healthcare provider while using my adherence technology	4.38	(4.14, 4.61)	4.38	(4.18, 4.58)	4.37	(4.12, 4.62)	4.41	(4.03, 4.78)
I am comfortable using my adherence technology in front of other people.	3.75	(3.38, 4.11)	4.01	(3.67, 4.35)	4.01	(3.69, 4.34)	3.64	(2.90, 4.39)
I am NOT worried that using the adherence technology will make it more likely that others will find out that I have TB.	3.78	(3.45, 4.12)	3.91	(3.50, 4.32)	3.89	(3.46, 4.28)	3.84	(3.03, 4.67)
It DOES NOT take me too much time to take my TB medicine and open the box every day.	4.35	(4.12, 4.57)	4.36	(4.19, 4.53)	4.32	(4.10, 4.54)	4.44	(4.09, 4.80)
I will recommend using adherence technologies to my family or friends if they have TB.	4.52	(4.33, 4.70)	4.60	(4.46, 4.75)	4.56	(4.37, 4.75)	4.59	(4.44, 4.75)
I am comfortable using the adherence technologies outside of my home (for example at work or when I travel).	3.95	(3.62, 4.27)	4.07	(3.72, 4.42)	4.02	(3.63, 4.41)	4.03	(3.39, 4.67)
I am comfortable taking the medication box to the clinic during my follow-up visit	4.34	(4.12, 4.56)	4.40	(4.23, 4.57)	4.34	(4.12, 4.55)	4.47	(4.17, 4.78)
**Motivation**	**4.37**	**(4.21, 4.53)**	**4.42**	**(4.27, 4.57)**	**4.39**	**(4.20, 4.58)**	**4.43**	**(4.18, 4.69)**
Using my adherence technology will help me complete my TB treatment.	4.51	(4.38, 4.64)	4.58	(4.45, 4.71)	4.54	(4.39, 4.69)	4.58	(4.39, 4.76)
Using adherence technologies while taking TB medicines will help me get healthy.	4.41	(4.22, 4.59)	4.54	(4.40, 4.68)	4.52	(4.35, 4.68)	4.42	(4.12, 4.72)
The instructions on the adherence technology makes it easy for me to remember what to do.	4.37	(4.17, 4.56)	4.38	(4.25, 4.51)	4.33	(4.16, 4.50)	4.49	(4.27, 4.71)
I am NOT concerned about the privacy of my health information collected by the adherence technology	4.20	(3.92, 4.48)	4.17	(3.86, 4.47)	4.16	(3.83, 4.49)	4.24	(3.60, 4.88)

Table S4a: Mean Capability, Opportunity, and Motivation scores by sex, age and treatment phase among participants using labels

	Sex				Age, grouped			
	Female (n=73)		Male (n=96)		<40 years (n=96)		≥40 years (n=73)	
	Mean	95% CI	Mean	95% CI	Mean	95% CI	Mean	95% CI
**Capability**	**4.32**	**(4.10, 4.54)**	**4.49**	**(4.37, 4.62)**	**4.48**	**(4.30, 4.65)**	**4.34**	**(4.19, 4.50)**
I know how to use the adherence technology	4.30	(3.97, 4.63)	4.49	(4.32, 4.67)	4.52	(4.26, 4.77)	4.27	(4.05, 4.49)
The reminders I receive on my phone help me to remember to take my medicine.	4.34	(4.08, 4.61)	4.49	(4.37, 4.61)	4.44	(4.24, 4.64)	4.41	(4.26, 4.56)
**Opportunity**	**4.19**	**(3.90, 4.49)**	**4.24**	**(4.02, 4.45)**	**4.32**	**(4.07, 4.58)**	**4.09**	**(3.84, 4.33)**
I am more connected to my healthcare provider while using my adherence technology	4.30	(4.04, 4.56)	4.36	(4.10, 4.63)	4.51	(4.27, 4.75)	4.11	(3.84, 4.37)
I am comfortable using my adherence technology in front of other people.	4.07	(3.65, 4.49)	4.23	(3.98, 4.48)	4.27	(3.94, 4.60)	4.01	(3.71, 4.31)
I am NOT worried that using the adherence technology will make it more likely that others will find out that I have TB.	4.07	(3.64, 4.50)	3.89	(3.41, 4.38)	4.08	(3.63, 4.54)	3.82	(3.41, 4.24)
It DOES NOT take me too much time to take my TB medicine and text for the labels every day.	4.07	(3.77, 4.37)	4.27	(3.95, 4.59)	4.27	(3.98, 4.56)	4.07	(3.77, 4.37)
I will recommend using adherence technologies to my family or friends if they have TB.	4.58	(4.30, 4.85)	4.60	(4.43, 4.78)	4.66	(4.45, 4.87)	4.51	(4.26, 4.75)
I am comfortable using the adherence technologies outside of my home (for example at work or when I travel).	4.08	(3.64, 4.52)	4.08	(3.75, 4.41)	4.15	(3.78, 4.51)	4.00	(3.59, 4.41)
**Motivation**	**4.34**	**(4.08, 4.60)**	**4.34**	**(4.20, 4.48)**	**4.39**	**(4.21, 4.58)**	**4.27**	**(4.04, 4.49)**
Using my adherence technology will help me complete my TB treatment.	4.44	(4.21, 4.68)	4.50	(4.33, 4.67)	4.52	(4.33, 4.70)	4.42	(4.19, 4.66)
Using adherence technologies while taking TB medicines will help me get healthy.	4.45	(4.22, 4.68)	4.48	(4.31, 4.65)	4.52	(4.33, 4.71)	4.40	(4.19, 4.61)
The instructions on the adherence technology makes it easy for me to remember what to do.	4.34	(4.14, 4.54)	4.39	(4.26, 4.51)	4.42	(4.27, 4.56)	4.30	(4.08, 4.52)
I am NOT concerned about the privacy of my health information collected by the adherence technology	4.11	(3.60, 4.62)	4.00	(3.67, 4.33)	4.13	3.74, 4.51)	3.95	(3.54, 4.35)

Table S4b: Mean Capability, Opportunity, and Motivation scores by sex, age and treatment phase among participants using labels

	Treatment phase				Area of residence			
	Intensive phase (n=50)		Continuation phase (n=119)		Urban (n=115)		Rural (n=54)	
	Mean	95% CI	Mean	95% CI	Mean	95% CI	Mean	95% CI
**Capability**	**4.39**	**(4.18, 4.60)**	**4.43**	**(4.28, 4.58)**	**4.45**	**(4.24, 4.66)**	**4.35**	**(4.21, 4.49)**
I know how to use the adherence technology	4.42	(4.11, 4.73)	4.41	(4.18, 4.63)	4.39	(4.10, 4.69)	4.44	(4.13, 4.76)
The reminders I receive on my phone help me to remember to take my medicine.	4.36	(4.10, 4.62)	4.45	(4.31, 4.60)	4.50	(4.35, 4.66)	4.26	(3.88, 4.64)
**Opportunity**	**4.22**	**(3.92, 4.52)**	**4.22**	**(3.98, 4.46)**	**4.25**	**(3.98, 4.52)**	**4.16**	**(3.56, 4.77)**
I am more connected to my healthcare provider while using my adherence technology	4.22	(3.92, 4.52)	4.39	(4.13, 4.64)	4.41	(4.15, 4.67)	4.19	(3.63, 4.74)
I am comfortable using my adherence technology in front of other people.	3.94	(3.48, 4.40)	4.25	(3.98, 4.53)	4.23	(3.90, 4.55)	4.02	(3.30, 4.74)
I am NOT worried that using the adherence technology will make it more likely that others will find out that I have TB.	4.20	(3.77, 4.63)	3.87	(3.44, 4.31)	3.97	(3.51, 4.44)	3.96	(3.00, 4.93)
It DOES NOT take me too much time to take my TB medicine and text for the labels every day.	4.20	(3.78, 4.62)	4.18	(3.92, 4.43)	4.17	(3.79, 4.54)	4.22	(3.84, 4.61)
I will recommend using adherence technologies to my family or friends if they have TB.	4.64	(4.39, 4.89)	4.57	(4.37, 4.78)	4.57	(4.28, 4.87)	4.63	(4.28, 4.98)
I am comfortable using the adherence technologies outside of my home (for example at work or when I travel).	4.12	(3.78, 4.46)	4.07	(3.67, 4.47)	4.14	(3.93, 4.35)	3.96	(2.79, 5.13)
**Motivation**	**4.28**	**(4.05, 4.52)**	**4.36**	**(4.18, 4.54)**	**4.32**	**(4.07, 4.56)**	**4.39**	**(4.06, 4.71)**
Using my adherence technology will help me complete my TB treatment.	4.33	(4.05, 4.60)	4.54	(4.35, 4.73)	4.48	(4.23, 4.73)	4.47	(4.33, 4.62)
Using adherence technologies while taking TB medicines will help me get healthy.	4.36	(4.08, 4.64)	4.51	(4.34, 4.69)	4.45	(4.22, 4.68)	4.50	(4.15, 4.85)
The instructions on the adherence technology makes it easy for me to remember what to do.	4.26	(4.02, 4.49)	4.41	(4.25, 4.57)	4.34	(4.14, 4.54)	4.43	(4.19, 4.66)
I am NOT concerned about the privacy of my health information collected by the adherence technology	4.18	(3.77, 4.59)	3.99	(3.63, 4.35)	4.00	(3.53, 4.47)	4.15	(3.60, 4.88)

Table S5: Mean Capability, Opportunity, and Motivation scores by facility type among participants in Tanzania and Philippines

	Participants in Tanzania using the pillbox				Participants in Philippines using the label			
	Private (n=20)		Public (n=41)		Private (n=10)		Public (n=42)	
	Mean[ǂ]	95% CI	Mean[ǂ]	95% CI	Mean[ǂǂ]	95% CI	Mean[ǂ]	95% CI
Capability	4.20	(0.81, 7.59)	4.28	(3.94, 4.62)	4.45	(3.99, 4.91)	4.19	(4.08, 4.30)
Opportunity	4.44	(2.35, 6.52)	4.08	(3.74, 4.42)	3.78	(3.41, 4.15)	3.77	(3.14, 4.40)
Motivation	4.49	(3.06, 5.92)	4.33	(4.13, 4.74)	4.20	(4.02, 4.38)	3.93	(3.58, 4.28)

[ǂ] Mean scores are adjusted for clustering [ǂǂ] Mean scores are calculated from one cluster
